# Macrofouling communities and the degradation of plastic bags in the sea: an *in situ* experiment

**DOI:** 10.1098/rsos.170549

**Published:** 2017-10-25

**Authors:** Nora-Charlotte Pauli, Jana S. Petermann, Christian Lott, Miriam Weber

**Affiliations:** 1 GEOMAR Helmholtz Centre for Ocean Research Kiel, Wischhofstr. 1-3, 24148 Kiel, Germany; 2 Institute of Biology, Freie Universität Berlin, Königin-Luise-Str. 1-3, 14195 Berlin, Germany; 3 Department of Ecology and Evolution, University of Salzburg, Hellbrunnerstrasse 34, 5020 Salzburg, Austria; 4 HYDRA Institute for Marine Sciences, Elba Field Station, Via del Forno 80, 57034 Campo nell'Elba (LI), Italy

**Keywords:** biodegradable plastic, polyethylene polymer, tensile properties, oxygen production, biodiversity, Mediterranean Sea

## Abstract

The increasing amount of plastic littered into the sea may provide a new substratum for benthic organisms. These marine fouling communities on plastic have not received much scientific attention. We present, to our knowledge, the first comprehensive analysis of their macroscopic community composition, their primary production and the polymer degradation comparing conventional polyethylene (PE) and a biodegradable starch-based plastic blend in coastal benthic and pelagic habitats in the Mediterranean Sea. The biomass of the fouling layer increased significantly over time and all samples became heavy enough to sink to the seafloor. The fouling communities, consisting of 21 families, were distinct between habitats, but not between polymer types. Positive primary production was measured in the pelagic, but not in the benthic habitat, suggesting that large accumulations of floating plastic could pose a source of oxygen for local ecosystems, as well as a carbon sink. Contrary to PE, the biodegradable plastic showed a significant loss of tensile strength and disintegrated over time in both habitats. These results indicate that in the marine environment, biodegradable polymers may disintegrate at higher rates than conventional polymers. This should be considered for the development of new materials, environmental risk assessment and waste management strategies.

## 1. Introduction

Plastic pollution has been an increasing problem in the oceans for the past five decades. Today, plastic makes up more than 80% of all marine debris and occurs throughout all marine habitats, from the deep ocean to the intertidal zones of beaches [1,2].

Once plastic litter has entered the sea [1], it undergoes different fates. Depending on its density, plastic will float at the surface, be neutrally buoyant, or sink. The density of lighter items will increase when they are colonized by fouling organisms, causing them to eventually sink [3]. Up to 94% of all plastic marine debris (PMD) is estimated to end up on the seafloor [4]. High amounts have also been discovered in intertidal and subtidal coastal sediments [2,5].

Plastics are synthetic polymers [6] and are composed of a large variety of different polymer types. Polyethylene (PE) is one of the most common polymers, making up 29.3% of the yearly plastic demand in 2014 in the European Union [7]. Thus it is also a common polymer in PMD [1]. Similar to most other synthetic polymers, PE is very resistant to degradation in marine *in situ* conditions [8]. Thus, measures to reduce the amount of PMD and the development of alternative degradable polymers have become important fields of research. For example, polymer blends based on corn starch (e.g. MaterBi®) are now used as a basis for carrier bags, which claim to be compostable and biodegradable [9]. MaterBi® was tested in and on marine coastal sediments in laboratory experiments where it was biodegraded by 90% after less than a year [10].

Degradation is an irreversible multi-stage process influenced by a variety of biotic and abiotic factors, which change the polymer structure and subsequently cause a loss of properties [11] (see the electronic supplementary material for definitions of different polymers).

So far, few field studies have investigated the degradation of plastic comparing different polymers. O'Brine & Thompson [12] tested different plastic bags in shallow waters (−0.6 m) of the North Sea for 40 days and showed faster degradation (loss of tensile properties and surface area) of biodegradable bags compared to PE bags. By contrast, no significant loss of tensile properties of a PE-starch blend [13] was found after 13 months of exposure to seawater in Long Island, USA, although a reduction of 32–34% of the starch content was measured. There is only one study available investigating sunken plastic on the sea floor [14].

As with every other unprotected solid surface in the marine environment, plastic will be colonized almost immediately after it has been submerged [3]. This colonization is called fouling and follows a specific sequence [15]. Bacteria and other microorganisms settle on the surface within a few hours, building a biofilm which provides cues for the settlement of larger organisms such as algal spores and the larvae of benthic invertebrates [16,17]. Because of ongoing littering the artificial surface area in marine habitats is continuously increasing [18]. Thus, the effects of fouling communities colonizing these surfaces on the surrounding ecosystem urgently need to be studied.

The biomass of the fouling community, as well as its composition, may be influenced by different surface characteristics such as wettability, roughness, or hydrophilic/hydrophobic properties [19]. Therefore, different polymers may be fouled at different rates and by distinct communities. Zettler *et al.* [20] investigated the microbial communities on marine plastic litter and suggested that there is a specific community which forms an ecologically diverse ‘plastisphere’. Microfouling communities on marine plastic debris are composed of archaea, fungi, bacteria, diatoms, dinoflagellates and coccolithophores among others [17,21]. Macrofouling communities are typically formed by bryozoans, barnacles (Crustacea), hydroids (Cnidaria) and polychaetes (Annelida) [22,23]. However, these macrofouling communities have rarely been studied and comparisons have rarely been made between the communities found on different polymer types, and the influence of the surrounding habitat. In addition, there are limited studies on the metabolic activity of plastic fouling communities, which are important for two reasons. Firstly, large accumulations of plastic in the water column may act as areas of oxygen production or consumption and thus influence the oxygen concentration in the surrounding water. Secondly, microbes associated with plastic, i.e. aerobic and facultative anaerobic microorganisms, may be involved in degradation processes of the polymer [24]. However, whether plastic is degraded best under aerobic or anaerobic marine *in situ* conditions has not yet been shown.

So far, few studies have combined measurements regarding fouling and degradation. Eich *et al.* [14] analysed the early stages of colonization on plastic carrier bags and found no significant difference in the diatom abundance between PE and MaterBi®. However, they found significantly different diatom community compositions between the two polymer types in a pelagic habitat. Additionally, the degradation of the two plastic types was investigated in terms of tensile strength and ultrastructure analysis by scanning electron microscopy. Although small holes were found after 33 days at 15°C, no reduction in tensile properties was observed. This suggests that degradation processes need to be studied over longer periods of time.

Here we present a 1 year *in situ* study with a comprehensive analysis of the structure of macrofouling communities, their primary production and polymer disintegration on two different polymer types in two coastal marine habitats (benthic versus pelagic). We investigated the biomass of the fouling layer, the composition of the macrofouling communities, their biodiversity, their oxygen production/consumption, as well as the disintegration and the tensile properties of MaterBi® and PE carrier bags. We aimed to test the following hypotheses: (i) the biomass of the fouling layer increases with time, (ii) the macrofouling communities and their structure are specific to the polymer type and to the habitat to which the polymer is exposed, (iii) the net primary production of the fouling community is positive in both habitats and higher in the pelagic habitat compared to the benthic habitat, and (iv) the biodegradable polymer disintegrates and loses tensile properties at similar rates in both habitats, while the polyethylene polymer does not show signs of disintegration and loss of tensile properties in either of the two habitats.

## 2. Material and methods

### 2.1. Study site and experimental design

This study was conducted from July 2013 to August 2014 off the island of Elba, Italy (42°43.617 N, 10°09.598 E) in the Mediterranean Sea. The conventional polyethylene plastic (PE) and a biodegradable polymer blend were exposed to a sublittoral (here called ‘benthic’) and a pelagic coastal habitat. The polymer samples were PE fruit and vegetable carrier bags (20 µm thickness) and MaterBi® carrier bags (22 µm thickness) from a local supermarket (for details see Tosin *et al*. [25]). MaterBi® is a biodegradable blend made of a partially biobased copolyester (monomers made of vegetable oils and corn starch). The composition of both polymers was analysed by Fourier-transform infrared spectroscopy (FTIR) and compared to samples of known composition (electronic supplementary material, figure S1). This analysis confirmed the used polymers to be PE and MaterBi®, respectively. Polymer films with a size of 276 cm^2^ were secured in plastic meshes of 1.27 × 1.27 cm to avoid the eventual loss of larger disintegrated fragments. These meshes were fixed to an experimental construction combining a pelagic and a benthic experimental system (electronic supplementary material, figure S2), which was deployed with five replicates. The pelagic systems were suspended from a float at 25 m in the water column (electronic supplementary material, figure S3*a*) and were fixed with a rope to an anchor weight. The benthic systems were deployed at 36 m depth, on a seafloor with medium coarse sandy sediment (electronic supplementary material, figure S3*b*) and secured with a rope to the same anchor weight. Mean salinity at the site was 38 and the average annual temperature was 19°C. Samples were collected sequentially from each replicate construction by SCUBA divers four times over the period of 1 year, in September 2013 and in January, April, and July 2014. Owing to fishery activities the experimental construction of one replicate was lost between September and January, a second replicate was lost between January and April.

### 2.2. Measurements

The biomass of the fouling layer was quantified using a modified protocol by Lobelle & Cunliffe [26]. The polymer samples were dried and stained with an aqueous solution of 1% crystal violet (Sigma). Pieces with an average size of 120 cm^2^ were cut off the samples, rinsed three times with water, dried again and transferred to 96% ethanol to extract the dye. The optical density (OD) of the dye extract was measured photometrically at 595 nm (HACH, ‘DREL 2400’) and OD m^−2^ was used as a proxy for the relative biomass of the fouling layer. The fouling layer was scraped off a second piece (average size 67 cm^2^) using a nylon brush and subsequently fixed in 90% ethanol after dehydration in an alcohol-water dilution series. Identification of the fouling organisms was conducted under a stereomicroscope (Stemi 2000-C, Zeiss) at a 50-fold magnification. To evaluate the fouling community, the richness, Shannon diversity and abundance (specimens per cm^2^) were calculated for each sample. Here, we present data on 21 taxa which were identified to family level, which was used for all further community analyses. Data of the taxa with lower taxonomic resolution were excluded from the analysis, but are available in a data package in the Dryad database.

To evaluate the net primary production of the fouling community, polymer pieces of 10 cm^2^ size were incubated for 12 h in the dark and six hours under light conditions (Biolux 36 W/965, 3024 lx) in the laboratory. Schott glass bottles (100 ml) were filled with oxygenated seawater. The samples were incubated in the closed bottles and oxygen and temperature were measured (WTW, Weilheim, Germany) before and after each incubation step. The net oxygen production was calculated as the difference between gross production and respiration. All data were corrected for temperature differences.

The mechanical properties of the samples were measured as a proxy for the degradation and change of the molecular structure of the polymers. Tensile strength at break reported in Newton (N) was measured with a dynamometer (Instron 5500 Series, USA) as described by Tosin *et al.* [25].

The disintegration of the plastic film was determined photogrammetrically as per cent of area loss: Wet samples were photographed with a SLR camera (EOS 5D, Canon Inc.) and analysed for the proportion of lost versus intact surface using the software ImageJ (https://imagej.nih.gov/ij/) and GIMP (http://www.gimp.org/).

### 2.3. Data analyses

Statistical analyses were conducted using the software R, v. 3.2.1 [27]. The effects of the explanatory variables habitat (benthic versus pelagic), (polymer) type and time, and their interactions on the response variables OD m^−2^ (fouling layer), Shannon diversity, abundance, family-level richness, net oxygen production, disintegration and tensile strength were tested using linear mixed effect models after testing the validity of the assumptions (normality and homogeneity of variance). To incorporate the structure of the nested design, we included three nested random effects in the mixed model analysis, namely the replicate construction (‘replicate’), the term ‘habnest’ to account for the two locations of the samples in the two habitats per replicate construction and the term ‘typenest’ to account for the samples of the two polymer types per habitat per replicate construction. Data are displayed with fitted curves and respective confidence interval (95%) using the method ‘loess’ (local polynomial regression fitting, ggplot2 package for R) of the respective five to three replicates per sampling time point. The community composition at family level was analysed using a permutational multivariate analysis of variance (PERMANOVA) with the vegan package in R [28]. The difference in the homogeneity of group dispersions was tested using the betadisper function of the same package.

## 3. Results

### 3.1. Fouling community

**Figure 1.**
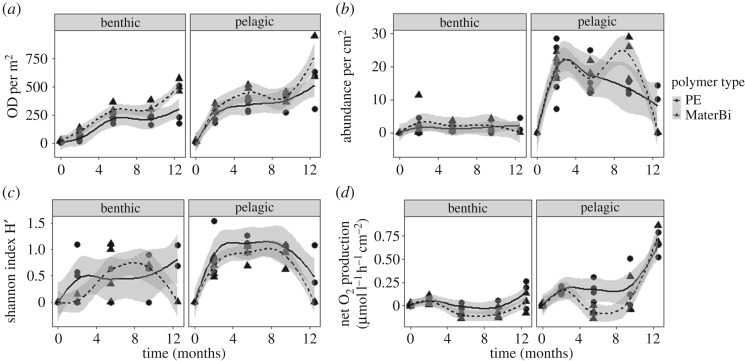
Measures of the fouling community structure and oxygen production changing over time. (*a*) Optical density (OD) per m^2^ as a proxy for the biomass of the fouling layer. (*b*) Abundance per cm^2^ for all macrofouling organisms identified to family level. (*c*) Shannon diversity based on the level of family. (*d*) Net oxygen production of the fouling community (µmol l^−1^ h^−1 ^cm^−2^). Results of the benthic habitat are shown on the left side, and of the pelagic habitat on the right side. Time is depicted in months of exposure. The curves and respective confidence interval (95%) were fitted using the method ‘loess’ in R.

Fouling increased over time in both habitats and on both polymers, but was 1.7-fold higher in the pelagic habitat compared with the benthic habitat and 1.3-fold higher on the biodegradable plastic in both habitats compared with PE (electronic supplementary material, table S1). In the fouling community, a total of 21 families were identified belonging to the phyla Foraminifera, Porifera, Cnidaria, Mollusca, Annelida, Nematoda and Bryozoa (electronic supplementary material, figures S4 and S5). Family richness differed significantly between the pelagic and the benthic habitats (*p* = 0.011; electronic supplementary material, table S2). The pelagic community consisted of 18 families, while the benthic community comprised 11 families (electronic supplementary material, figure S6). On the family level, Foraminifera were the most diverse phylum (10 families, six of them benthic, nine pelagic). Overall, abundances were higher in the pelagic community (*p* = 0.006; figure 1*b*; electronic supplementary material, table S3).

Biodiversity (Shannon diversity) increased over time (*p* = 0.005; figure 1*c*; electronic supplementary material, table S4) and was higher in the pelagic than in the benthic community (*p* = 0.045). The sudden decrease in biodiversity from 9.5 to 12.5 months on the biodegradable plastic polymer coincides with the loss of two replicates constructions, and the onset of increased disintegration of the samples. However, the diversity of the community on the PE samples decreased in the respective time period as well.

The fouling communities differed in composition between both habitats (PERMANOVA, *p* = 0.0018; electronic supplementary material, table S5). An analysis of the group dispersion showed that the pelagic communities were more variable in comparison to the benthic communities, which were more similar to each other (*p* < 0.001, average distance to mean: benthic = 1.49, pelagic = 5.9; electronic supplementary material, figure S7). There was no difference in the dispersion of the communities between the plastic types nor over the duration of the study.

The biomass of the fouling layer, determined using the OD as a proxy, increased significantly over the period of 12.5 months (*p* < 0.0001; figure 1*a*; electronic supplementary material, table S6). The biomass of the fouling layer was influenced by the interactions between habitat and time (*p* = 0.031), as well as by the interactions between polymer type and time (*p* = 0.012). Including abundance as a covariable in the analysis of the fouling biomass resulted in a loss of the significant influence of the habitat (electronic supplementary material, table S7). The effect of the polymer however remained significant.

### 3.2. Metabolic activity

The net oxygen production of the fouling community increased in both habitats and on both polymer types over time (*p* < 0.001; figure 1*d*; electronic supplementary material, table S8) and differed significantly between the two habitats (*p* = 0.027). In the benthic habitat, it was negative or only slightly positive (mean = 0.01 µmol l^−1^ h^−1^ cm^−2^, s.e. = 0.01) on PE samples, and lower and mostly negative on the biodegradable samples (mean = −0.0101 µmol l^−1^ h^−1^ cm^−2^, s.e. = 0.02). In the pelagic habitat, net oxygen production on PE was positive during the entire experiment with an increasing trend over time (mean = 0.17 µmol l^−1^ h^−1^ cm^−2^, s.e. = 0.04), and lower on the biodegradable plastic with negative values after five months. On both plastic types in the pelagic domain there was a peak in net oxygen production after 12.5 months in July.

### 3.3. Material properties

**Figure 2.**
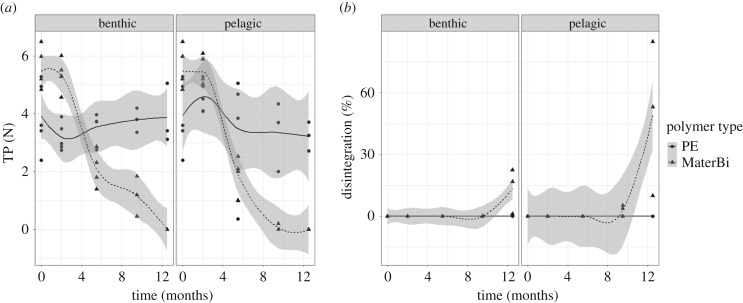
(*a*) Tensile properties (TP) and (*b*) disintegration of both polymer types in the benthic and pelagic habitat. Disintegration was measured as surface area loss in per cent. The curves and respective confidence interval (95%) were fitted using the method ‘loess’ in R. The number of replicates was reduced after 12.5 months owing to high fragmentation of the polymer.

**Figure 3.**
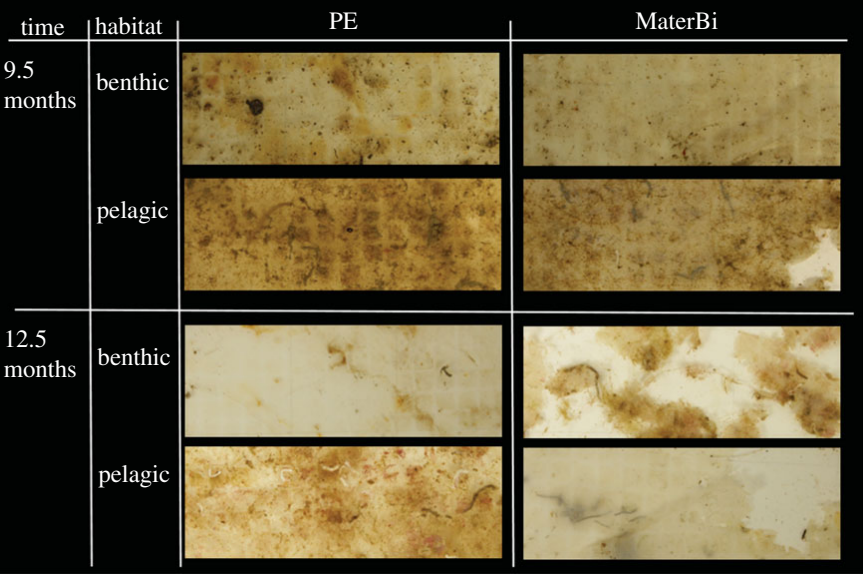
Disintegration of the PE carrier bag (left) and the biodegradable starch-based carrier bag (right) samples exposed to the benthic and pelagic habitat. After 9.5 months of exposure the biodegradable plastic showed first signs of brittleness and cracks, while no disintegration was visible in the PE samples. After 12.5 months of exposure the biodegradable plastic showed strong brittleness in the benthic habitat and was largely fragmented in the pelagic habitat, while no disintegration was visible in the PE samples.

Although PE has a specific weight lower than one, when processed in the laboratory in a water bath all benthic samples exposed for 5.5–12.5 months and all pelagic samples exposed for 2–12.5 months were heavy enough to sink to the bottom. Tensile strength of the biodegradable plastic samples declined over time in both habitats. By contrast, there was no significant loss of tensile strength of the PE samples over time, which is reflected in the significant interaction of time and type (*p* < 0.001; figure 2*a*; electronic supplementary material, table S9). In both habitats, tensile strength of the biodegradable plastic decreased between 2 and 5.5 months. Disintegration of the polymer was quantifiable on samples which were exposed to the field for at least 9.5 months (figure 2*b*). The loss of surface area indicates a higher degradation of the biodegradable plastic compared with PE. The same trend was observed for both of the habitats. However, disintegration of the biodegradable plastic was higher in the pelagic habitat (9.5 months: 3.01%, s.e. = 1.74%, 12.5 months: 49.23%, s.e. = 28.42%) than in the benthic habitat (9.5 months: 0.05%, s.e. = 0.03%, 12.5 months 13.44%, s.e. = 7.76%; figure 3). Means and standard errors for all response variables and habitat–polymer type combinations are available in the electronic supplementary material, table S1.

## 4. Discussion

### 4.1. The biomass of the fouling layer

In both habitats and on both polymer types the biomass of the fouling layer increased significantly over time, supporting our first hypothesis and confirming results by Lobelle & Cunliffe [26]. Statistical analyses of our results indicate that the habitat influenced biomass via changes in the abundance of the organisms and that the effect of the polymer was also apparent via changes in biomass per individual.

In a natural scenario an increasing fouling biomass on a plastic item, as found in the present study, would eventually lead to negative buoyancy of the item, causing it to sink [3]. This was not possible in our fixed experimental samples, but supports the notion that the majority of PMD is located on the seafloor [4]. These findings on the buoyancy of plastic influenced by fouling are important for the modelling of waste streams in the ocean. Further investigations will increase the understanding of the fate of plastic in the ocean allowing better estimations of the quantity of PMD in different habitats [4] and possibly help to identify areas for the removal of PMD [29].

### 4.2. The fouling communities and their structure

The families identified from our samples represent phyla typically known as macrofouling organisms on plastic and other artificial substrates [30]. Communities were not distinct between polymer types, rejecting our second hypothesis. In contrast to previous studies [22,31] surface characteristics of the polymers seemed less important for the biomass of the fouling layer. Other studies, although dealing with microfouling communities, support our findings: biofilm-forming bacteria and diatom communities show similar community compositions across different substrates as reviewed by Salta *et al.* [32]. Moreover, the identified taxa are common epibionts on the seagrass *Posidonia oceanica*, the predominant macrophyte at the study site [33]. These results support the notion that the majority of epibionts may be generalists in their choice of substratum [19].

On a higher taxonomic level the identified community structure presented here is comparable to that presented in studies from different parts of the global ocean (Mediterranean Sea [17,34], Indian Ocean around India [22], Indian/Pacific Ocean around Australia [21]). However, on a species level, diversity and relative abundances may vary widely [32].

Differences in community composition among habitats, such as those shown in this study, are of major importance in regard to the location of plastic accumulations in the ocean (see §4.1) and the respective impact on local ecosystems (see §4.3).

Our results showed a higher diversity in the pelagic compared to the benthic habitat. If the samples deployed in the pelagic habitat are considered a benthic habitat in their own right, this supports the general assumption that shallow benthic habitats have a higher diversity [35]. The higher ‘pelagic’ abundances may be attributed to higher light intensities at 25 m (pelagic samples) compared to light conditions at 36 m (benthic samples). Moreover, the availability of larvae and nutrients transported via currents is higher in the water column [36]. The month of exposure is an important factor influencing the successional stages of fouling communities [37,38]. Future studies investigating fouling communities on PMD should include genetic methods to achieve higher taxonomic resolution and combine data on macroscopic and microbial fouling organisms. In addition, a series of samples deployed at different seasons would account for seasonal variability.

### 4.3. The metabolic activity of the fouling community

Our data showed a positive net oxygen production in the pelagic habitat for the PE polymer during the entire experiment, whereas in the benthic zone negative production was recorded after five and nine months of exposure. This result partly confirms our third hypothesis that the net oxygen production is higher in the pelagic habitat. It is also in line with findings by Eich *et al*. [14], even though they investigated samples exposed for only 30 days. We observed a peak in net oxygen production after 12.5 months in July on both plastic types in the pelagic domain. This could be a matter of season, as light availability is the driving force of photosynthetic production.

Our results can be interpreted from two perspectives: (i) the effect on the surrounding ecosystem, and (ii) the effect on the degradation process.

(i) Assuming 400 million pieces of macroplastic (greater than 20 cm^2^) with a surface area of 1.6 km^2^ in the Mediterranean Sea [39], the contribution of communities on these plastic items to primary production is negligible when compared to the water surface area of the Mediterranean Sea (2.5 × 10^6^ km^2^). On a smaller scale, however, when plastic accumulates in certain areas, e.g. at the coast, this may be different. The average pelagic *net* oxygen production in our study was 0.12 µmol l^−1^ h^−1^ cm^2^ which equals the *gross* primary production in the Mediterranean Sea at comparable irradiance [40]. Thus, we assume that the *gross* production on the plastic surface is higher than that of the surrounding water and therefore constitutes an additional source of oxygen. Besides, large accumulations of plastic could account for a certain proportion of carbon export in the respective areas, as fouled plastic will sink with increasing weight.
(ii) The ratio of production and respiration of the fouling community on the PE samples was nearly zero, suggesting a constant remineralisation of the produced organic matter. On the biodegradable plastic, the ratio was negative. One possible explanation is that the polymer was remineralized. As the budget was only slightly negative, we assume that the remineralisation occurred at a rather low rate. Filamentous algae, diatoms, and dinoflagellates that were also found on the samples and which are most likely major primary producers within the fouling community have previously reported as microfouling organisms [17,41]. Unfortunately, these groups could not be included in the analysis owing to low taxonomic resolution. Only heterotrophic organisms could be identified with sufficient resolution.

### 4.4. Disintegration and tensile properties

The tested biodegradable plastic showed disintegration and significant loss of tensile properties over time suggesting that bags based on this polymer will disintegrate under similar conditions (see the electronic supplementary material for method discussion). This supports our fourth hypothesis and coincides with results presented by O'Brine & Thompson [12], who exposed MaterBi® carrier bags in surface waters (−0.6 m) in Plymouth, UK. However, both field studies lack the direct proof of *bio*degradation. Contrary to simple degradation, which leads to disintegration, i.e. the breaking-up of an item into smaller fragments, *bio*degradation is caused by organismic activity [11] leading to a complete transformation of all components into CO_2_ or CH_4_, water and biomass. While field studies can only give results on general degradation [8], true *bio*degradation can only be measured in closed systems in the laboratory. Therefore, it is mandatory to confirm and complete field studies with laboratory tests measuring CO_2_ evolution or O_2_ consumption in a closed container as proposed by Ratto *et al*. [42]. MaterBi® was tested in such a container under optimized marine benthic conditions (constant temperature, addition of nutrients) [10,25] and showed signs of disintegration, as well as biodegradation. However, biodegradation in the field might take longer than under optimized laboratory conditions.

Contrary to the biodegradable polymer, the PE polymer showed no change in tensile strength or disintegration in this study confirming results from the Bay of Plymouth, UK [12], the Baltic Sea and laboratory studies [43] for exposure times of 40 weeks and 20 months. Contrasting results were reported from the Bay of Bengal, India where LDPE, HDPE and PET showed weight loss of 0.5 to 7.49% after six and 12 months [22,23,31]. In laboratory tests bacterial degradation led to a weight loss of 8% of PE within 30 days [44].

## 5. Conclusion

Our results indicate that fouling communities are similar on PMD regardless of the polymer type and that it will probably sink to the seafloor. The biodegradable polymer blend tested here disintegrated at much higher rates than the PE polymer in benthic and pelagic habitats. Thus, it might remain in the sea for shorter times than PE and could pose a lower risk for marine organisms and ecosystems. We therefore see an obvious need to establish sustainable biodegradable alternatives to conventional polymers like PE, especially for applications where discard into the natural environment is likely. Our results indicate how the fate and degradation of plastic polymers in the marine environment may be influenced by fouling communities, as well as how the metabolic activity of the fouling community might influence the surrounding water. Future studies should continue investigating the combinational effects of biofilm formation, fouling communities, its metabolic activity and degradation processes in other marine habitats, such as beaches, muddy shallow-water seafloors and the deep sea, as well as under eutrophic and anoxic conditions. Moreover, we suggest verifying the disintegration under field conditions and studying the impact of new materials prior to their introduction to the environment from a cellular to an ecosystem level, including studies on ecotoxicological aspects.

## Supplementary Material

Supplementary material

## Supplementary Material

Dataset on fouling organisms and measurements

## Data accessibility

All relevant data are available within the paper and its electronic supplementary material. All data can be accessed through the Dryad database at http://dx.doi.org/10.5061/dryad.n8274 [45].
