# Supplementary material for: Macrofouling communities and the degradation of plastic bags in the sea: an in situ experiment
Source: R Soc Open Sci. 2017 Oct 25;4(10):170549. doi: 10.1098/rsos.170549 (PMC5666253; doi:10.1098/rsos.170549)
Supplement: Supplementary material [file rsos170549supp1.pdf]

Electronic supplementary material

Macrofouling communities and the degradation of plastic bags in the sea – an *in situ* experiment

Nora-Charlotte Pauli[1,2], Jana S. Petermann[2,3], Christian Lott[4], Miriam Weber[4, *]

[1] GEOMAR Helmholtz Centre for Ocean Research Kiel, Wischhofstr. 1-3, 24148 Kiel, Germany

[2] Institute of Biology, Freie Universität Berlin, Königin-Luise-Str. 1-3, 14195 Berlin, Germany

[3] Department of Ecology and Evolution, University of Salzburg, Hellbrunnerstrasse 34, 5020 Salzburg, Austria

[4] HYDRA Institute for Marine Sciences, Elba Field Station, Via del Forno 80, 57034 Campo nell'Elba (LI), Italy

* Corresponding author: m.weber@hydra-institute.com

1. Introduction

There is a variety of sometimes misleading terms for different types of plastics, therefore we focused on three terms in our study: biodegradable, biobased and conventional plastic. *Bio*degradable plastics are degradable to full remineralisation (CO_2 or CH_4, water and biomass), and can be fossil- as well as biobased. Bio*based* plastic products consist of biogenic base materials such as starch or vegetable oils. They can be, but are not necessarily, biodegradable. We refer to all synthetic products which are degradable only at very low rates as conventional polymers.

2. Material and Methods

Plastic bags made of two different polymer types, conventional polyethylene (PE) and a biodegradable starch-based polymer blend, were exposed to a benthic and a pelagic coastal habitat. The composition of the two polymers was analysed by Fourier-transform infrared spectroscopy (FTIR) and confirmed to be PE and MaterBi® respectively (Fig. S1).

There were five replicate experimental constructions, each consisted of a pelagic and a benthic system (Fig. S2), which were deployed at 25 m depth in the water column and at 36 m depth on a sediment floor, respectively (Fig. S3).

4. Discussion

4.4 Disintegration and tensile properties

The determination of degradation was conducted by following the loss of sample surface over time photogrammetry. This method, however, intrinsically leads to an underestimation of disintegration at the beginning of the study because a reduction of thickness of the foil is not accounted for as the

images are only two-dimensional. Only when the thinning of the foil leads to the formation of holes, a "loss of surface" will be recorded (Fig. 3). Thus, three-dimensional disintegration is likely to have started before the second half of the experiment. At a very late stage in the study the method tends to overestimate the degradation because fragments are likely to be lost from the mesh bags.

List of supplementary figures and tables

Figure S1. FTIR analysis of a biodegradable starch-based polymer blend (MaterBi) and a conventional polyethylene (PE).

Figure S2. Schematic sketch of the experimental construction of one replicate.

Figure S3. Pictures of the pelagic and benthic replicate construction *in situ*.

Figure S4. Abundances of all phyla of the fouling communities in the benthic and pelagic habitat.

Figure S5. Selected fouling organisms identified from the polymer samples.

Figure S6. Average abundances per cm² per family in the benthic and pelagic habitats.

Figure S7. Results of the analysis of homogeneity of multivariate dispersions.

Table S1. Means and standard errors for all response variable and all habitat-polymer type combinations.

Table S2. Results of the mixed model analysis of the effect of habitat, polymer type, time, and their interactions on family richness.

Table S3. Results of the mixed model analysis of the effect of habitat, polymer type, time, and their interactions on abundances of all specimens identified to family level.

Table S4. Results of the mixed model analysis of the effect of habitat, polymer type, time, and their interactions on Shannon diversity on family level.

Table S5. Results of the PERMANOVA analysis of the effect of habitat, polymer type, time, and their interactions on the composition of the fouling community identified to family level.

Table S6. Results of the mixed model analysis of the effect of habitat, polymer type, time, and their interactions on optical density (OD) as a proxy for the biomass of the fouling layer.

Table S7. Results of the mixed model analysis of the effect of habitat, polymer type, time, and their interactions on optical density (OD) as a proxy for the biomass of the fouling layer, with abundance on family level tested as a covariable.

Table S8. Results of the mixed model analysis of the effect of habitat, polymer type, time, and their interactions on net oxygen production.

Table S9. Results of the mixed model analysis of the effect of habitat, polymer type, time, and their interactions with tensile strength.

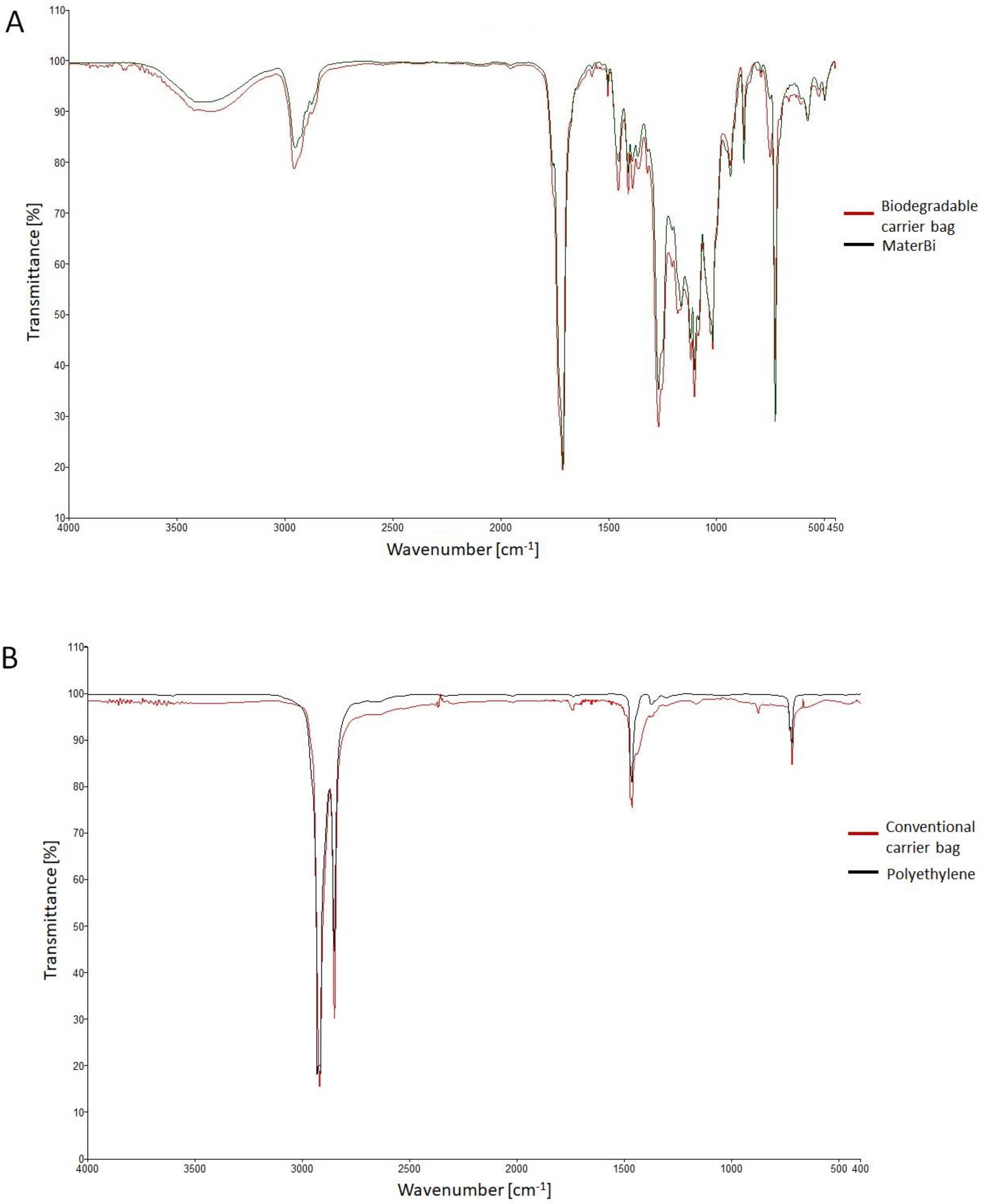

Figure S1: Results of the Fourier-transform infrared spectroscopy (FTIR) used to analyse the polymer composition. A: FTIR analysis of the biodegradable carrier bag assumed to be MaterBi®. B: FTIR analysis of the conventional fruit and vegetable carrier bag assumed to be Polyethylene (PE). The samples are shown in red, and compared to a polymer of known composition, shown in black. Wavenumber [cm^{-1}] is depicted on the x-axis. Transmittance of the sample is given in percent and is depicted on the y-axis.

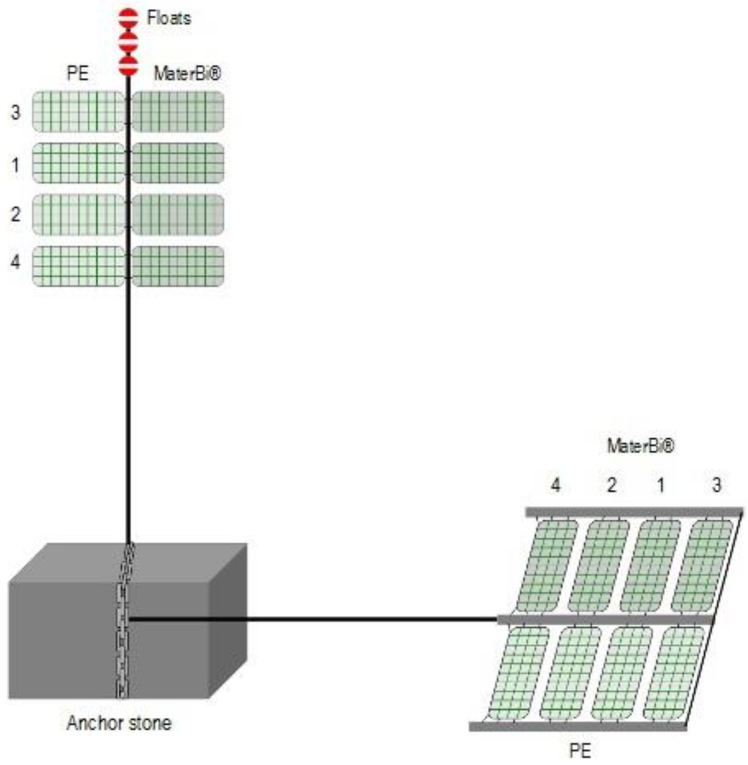

Figure S2. Schematic sketch of the experimental construction of one replicate. There were 5 replicate constructions in total. Plastic films of carrier bags made of Polyethylene (PE) and a biodegradable starch-based plastic blend were secured in mesh bags to avoid the eventual loss of disintegrated fragments. The mesh bags were attached to a benthic and a pelagic experimental system, both connected by ropes to an anchor stone on the seafloor. The benthic system was deployed on the sediment sea floor at 36 m depth. The pelagic system was suspended at 25 m depth in the water column and held upright by floats. On each side of the benthic and pelagic system four samples of the two polymer types were mounted. The numbers represent the pattern in which the samples were retrieved (1=2 months, 2=5.5months, 3=9.5months, 4=12.5 months).

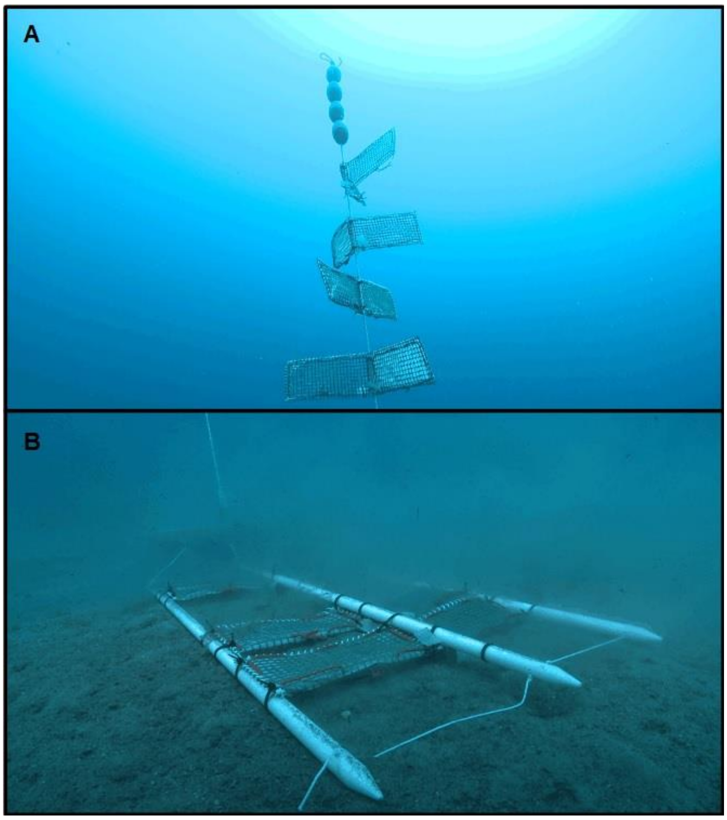

Figure S3. Pictures of the pelagic (A) and benthic (B) replicate construction *in situ*. Benthic samples were connected to an anchor stone at a depth of 36 m. Pelagic samples were floating in the water column at 25m, kept upright by floats.

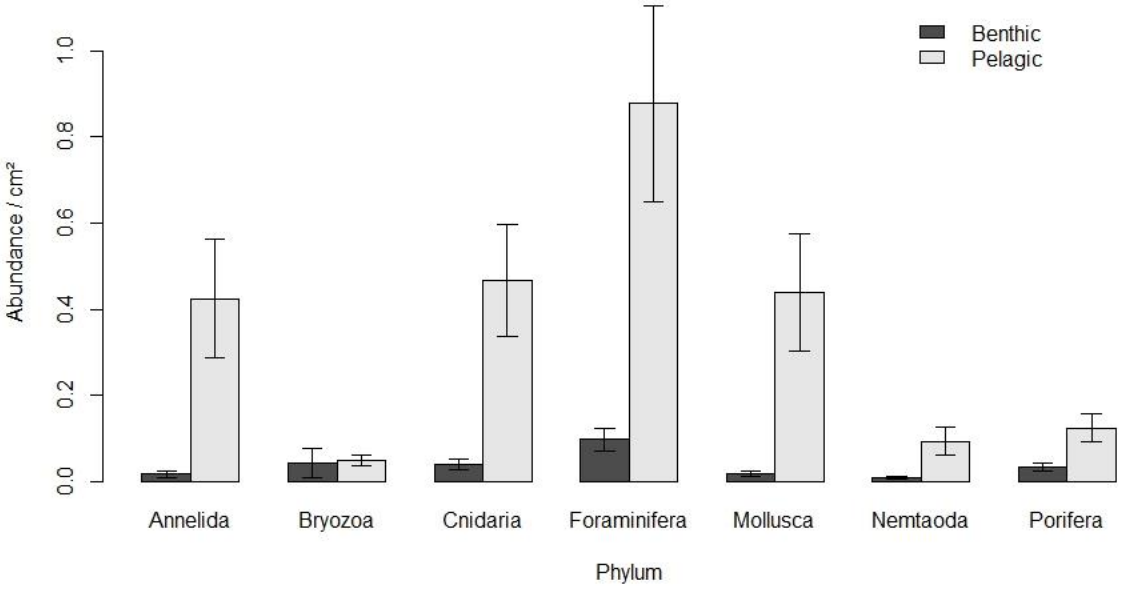

Figure S4. Abundances (number of specimens per cm²) of all phyla of the fouling communities in the benthic (dark grey) and pelagic (light grey) habitats. Averages across all samples and all exposure times with standard errors are shown.

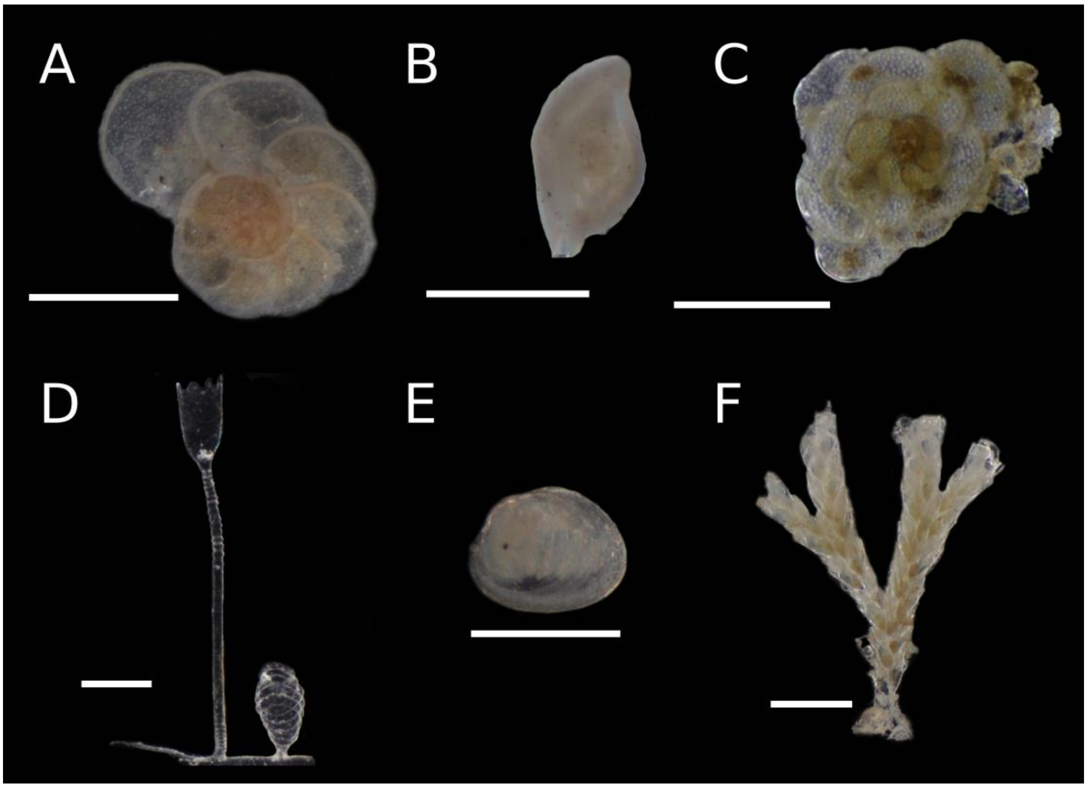

Figure S5. Selected fouling organisms identified from the polymer samples. A Trochammindae, Foraminifera, B Spiroloculinidae, Foraminifera, C Planorbulinidae, Foraminifera, D Campanulariidae, Cnidaria, E Mytilidae, Mollusca, F Candidae, Broyzoa. The white bars represent 0.5 mm for A-F, and 1 mm for F.

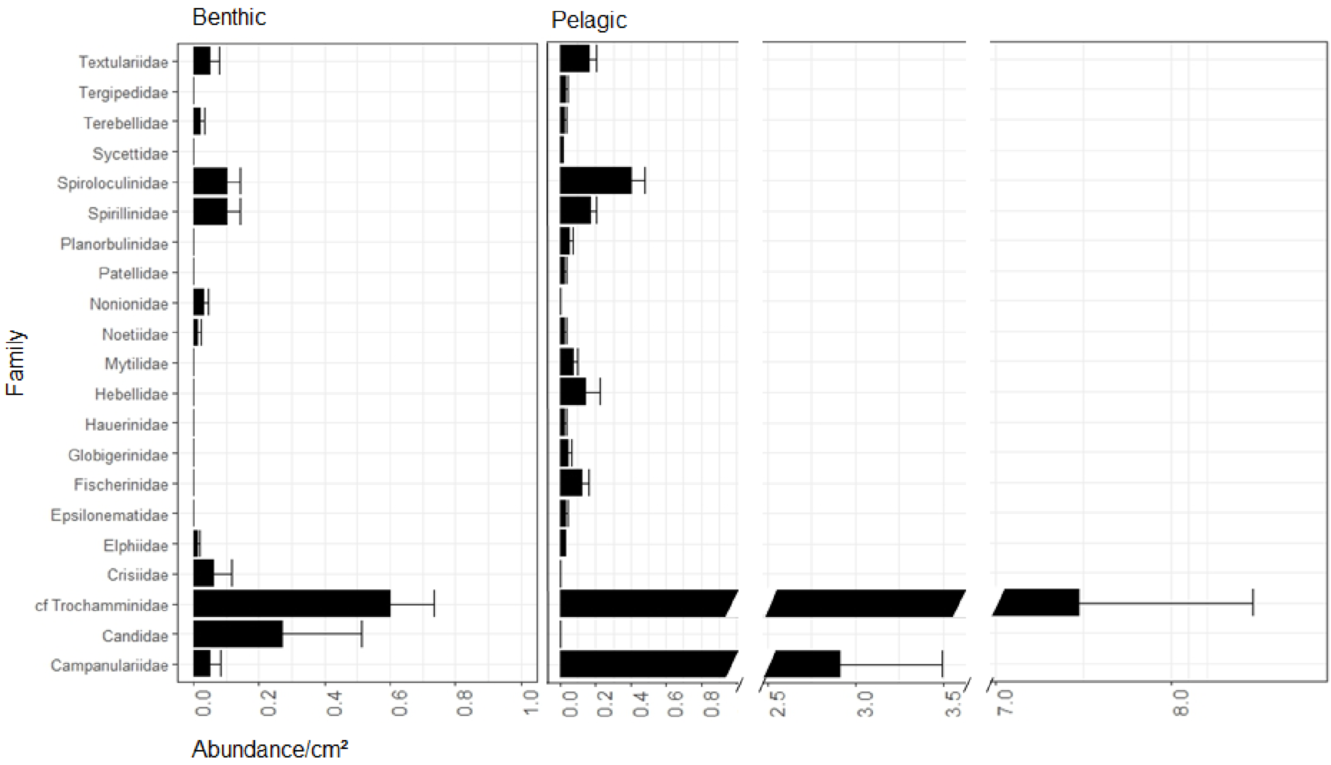

Figure S6: Average abundances per cm² per family in the benthic (left) and in the pelagic habitat (right). The average abundance of each of the 21 families identified in the fouling community is depicted with the respective standard error.

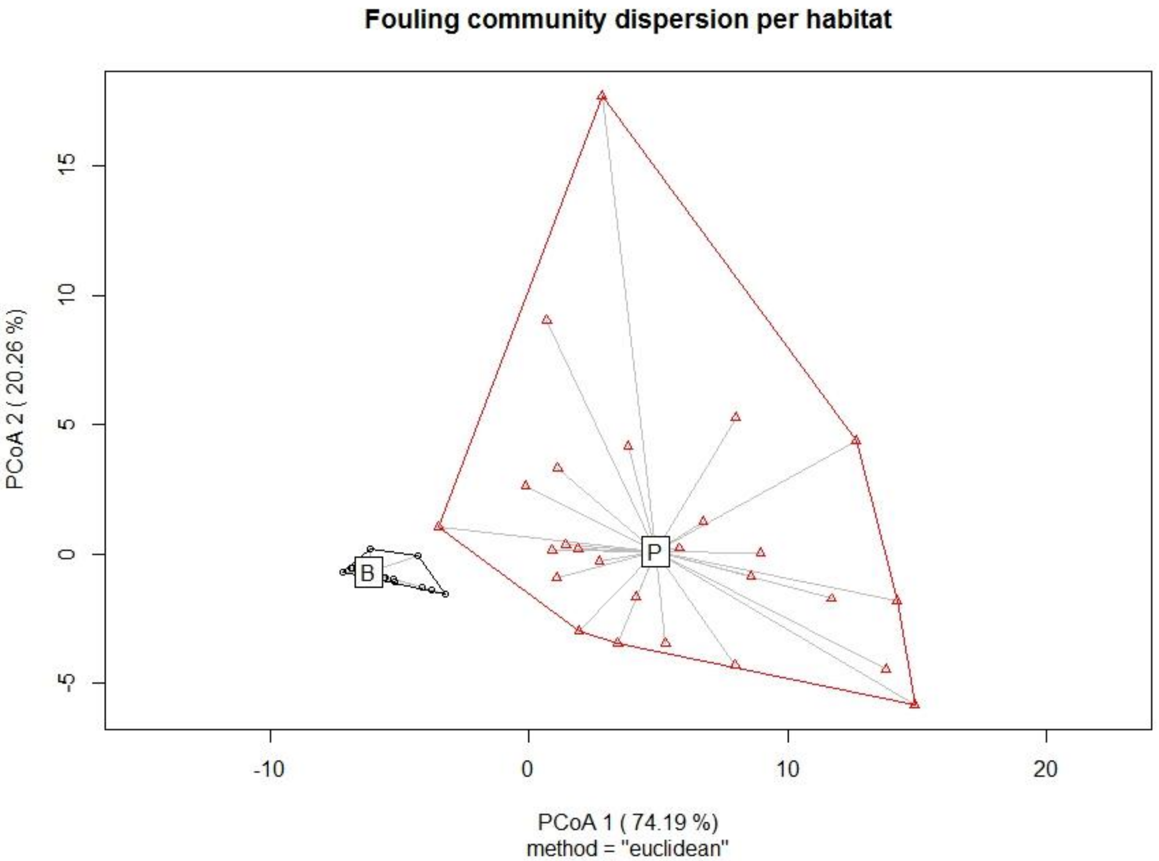

Figure S7: Results of the analysis of homogeneity of multivariate dispersions. The communities in the two habitats (benthic *vs*. pelagic.) were compared. The average distance of each group to the spatial median (=centroid) in a multivariate space was used as a measure for the dispersion (variance) of the groups. Euclidean distances were calculated based on the principal coordinate axes (PCoA). Eigenvalues are given in percent. The two habitats are depicted in red (pelagic, "P") and black (benthic, "B").

Table S1. Means and standard errors (se) for all response variables and all habitat - polymer type combinations. The two polymer types (conventional PE *vs*. biodegradable plastic) were exposed to a benthic and a pelagic coastal marine habitat. The fouling biomass (measured as optical density (OD) of the stained samples per m²), abundances (specimens per cm²), family richness, Shannon diversity on family level, net oxygen production [µmol L^{-1} h^{-1} cm^{-2}] of the fouling community and tensile strength of the polymers at break [N] were measured.

Response variable	Habitat	Polymer type	Mean	se
Fouling biomass	Benthic	PE	136.20	29.23
		biodegradable plastic	215.20	38.88
	Pelagic	PE	266.60	42.06
		biodegradable plastic	327.20	54.45
Abundance	Benthic	PE	1.17	0.33
		biodegradable plastic	1.45	0.59
	Pelagic	PE	11.55	2.10
		biodegradable plastic	11.84	2.38
Family richness	Benthic	PE	1.40	0.28
		biodegradable plastic	1.00	0.24
	Pelagic	PE	3.40	0.56
		biodegradable plastic	3.10	0.65
Shannon diversity	Benthic	PE	0.35	0.10
		biodegradable plastic	0.23	0.08
	Pelagic	PE	0.68	0.11
		biodegradable plastic	0.49	0.10
Net oxygen production	Benthic	PE	0.03	0.02
		biodegradable plastic	-0.01	0.02
	Pelagic	PE	0.21	0.05
		biodegradable plastic	0.12	0.06
Tensile strength	Benthic	PE	3.64	0.17
		biodegradable plastic	3.29	0.52
	Pelagic	PE	3.81	0.28
		biodegradable plastic	3.28	0.57

Table S2: Results of mixed model analysis of the effect of habitat, polymer type, time, and their interactions on family richness. Three nested random effects were included to incorporate the structure of the nested design: 'Replicate' for the replicate construction (combined benthic and pelagic experimental systems), 'habnest' to account for the two samples per habitat (benthic vs. pelagic) and 'typenest' to account for the two samples of the two polymer types (PE vs. biodegradable plastic) per habitat.

Family richness	numDF	denDF	F-value	p-value
(Intercept)	1	56	92.88	<0.0001
Habitat	1	4	19.71	0.0113
Type	1	8	0.58	0.4702
Time	1	56	5.44	0.0233
Habitat :Type	1	8	0.01	0.9164
Habitat : Time	1	56	0.33	0.5701
Type : Time	1	56	0.33	0.5685
Habitat : Type : Time	1	56	0.09	0.7707

Table S3: Results of mixed model analysis of the effect of habitat, polymer type, time, and their interactions on abundance for all specimens which were identified to family level. The abundance data were square root transformed to adjust for a non-normal distribution. Three nested random effects were included to incorporate the structure of the nested design: 'Replicate' for the replicate construction (combined benthic and pelagic experimental systems), 'habnest' to account for the two samples per habitat (benthic vs. pelagic) and 'typenest' to account for the two samples of the two polymer types (PE vs. biodegradable plastic per habitat).

Abundance	numDF	denDF	F-value	p-value
(Intercept)	1	56	94.28	<0.0001
Habitat	1	4	28.20	0.006
Type	1	8	0.06	0.010
Time	1	56	2.36	0.130
Habitat :Type	1	8	0.03	0.872
Habitat : Time	1	56	0.16	0.692
Type : Time	1	56	1.12	0.295
Habitat : Type : Time	1	56	0.02	0.882

Table S4: Results of mixed model analysis of the effect of habitat, polymer type, time, and their interactions on the Shannon diversity on family level. Three nested random effects were included to incorporate the structure of the nested design: 'Replicate' for the replicate construction (combined benthic and pelagic experimental systems), 'habnest' to account for the two samples per habitat (benthic vs. pelagic) and 'typenest' to account for the two samples of the two polymer types (PE vs. biodegradable plastic) per habitat.

Shannon diversity	numDF	denDF	F-value	p-value
(Intercept)	1	56	88.78	<0.0001
Habitat	1	4	8.19	0.0459
Type	1	8	3.43	0.1011
Time	1	56	8.73	0.0046
Habitat :Type	1	8	0.01	0.9285
Habitat : Time	1	56	0.43	0.5146
Type : Time	1	56	1.54	0.2203
Habitat : Type : Time	1	56	0.00	0.9947

Table S5: Results of the PERMANOVA analysis (999 permutations) of the effect of habitat, polymer type, time, and their interactions on the fouling community identified to family level (21 families, taxa that could not be identified to family level were excluded from this analysis). Pairwise Bray-Curtis distances were used.

Fouling community (families)	Df	Sums of Sqs	Mean Sqs	F-value	R^2	p-value
Habitat	1	5.44	5.44	32.94	0.4	0.001
Type	1	0.14	0.14	0.87	0.01	0.465
Time	1	0.27	0.27	1.63	0.02	0.159
Habitat :Type	1	0.11	0.11	0.64	0.01	0.669
Habitat : Time	1	0.23	0.23	1.39	0.02	0.179
Type : Time	1	0.22	0.22	1.31	0.02	0.227
Habitat : Type : Time	1	0.32	0.32	1.92	0.02	0.096
Residuals	42	6.93	0.17		0.51	
Total	43	13.65			1	

Table S6: Results of mixed model analysis of the effect of habitat, polymer type, time and their interactions on optical density (OD) as a proxy for the biomass of the fouling layer. Three nested random effects were included to incorporate the structure of the nested design: 'Replicate' for the replicate construction (combined benthic and pelagic experimental systems), 'habnest' to account for the two samples per habitat (benthic vs. pelagic) and 'typenest' to account for the two samples of the two polymer types (PE vs. biodegradable plastic) per habitat.

OD	numDF	denDF	F-value	p-value
(Intercept)	1	55	509.99	<0.0001
Habitat	1	4	33.44	0.004
Type	1	8	11.28	0.010
Time	1	55	219.37	<0.0001
Habitat :Type	1	8	0.01	0.918
Habitat : Time	1	55	4.93	0.031
Type : Time	1	55	6.72	0.012
Habitat : Type : Time	1	55	0.00	0.980

Table S7: Results of mixed effect model analysis of the effect of habitat, polymer type, time, and their interactions on optical density (OD). In contrast to Table S1, abundance on family level was tested as a covariable here in order to assess whether the effects of the explanatory variables on OD operated via abundance. Three nested random effects were included to incorporate the structure of the nested design: 'Replicate' for the replicate construction (combined benthic and pelagic experimental systems), 'habnest' to account for the two samples per habitat (benthic vs. pelagic) and 'typenest' to account for the two samples of the two polymer types (PE vs. biodegradable plastic) per habitat.

OD + Abundance	numDF	denDF	F-value	p-value
(Intercept)	1	54	542.52	<0.0001
Abundance	1	54	56.23	<0.0001
Habitat	1	4	3.16	0.1503
Type	1	8	10.63	0.0115
Time	1	54	217.66	<0.0001
Habitat :Type	1	8	0.03	0.8636
Habitat : Time	1	54	4.04	0.0494
Type : Time	1	54	7.12	0.0100
Habitat : Type : Time	1	54	0.01	0.9198

Table S8. Results of mixed effect model analysis of the effect of habitat, polymer time and type, and their interactions with net oxygen production. Three nested random effects were included to incorporate the structure of the nested design: 'Replicate' for the replicate construction (combined benthic and pelagic experimental systems), 'habnest' to account for the two samples per habitat (benthic vs. pelagic) and 'typenest' to account for the two samples of the two polymer types (PE vs. biodegradable plastic) per habitat.

Net oxygen production	numDF	denDF	F-value	p-value
(Intercept)	1	29	281.38	<0.0001
Habitat	1	4	11.57	0.0272
Type	1	8	0.12	0.7366
Time	1	29	32.06	<0.0001
Habitat :Type	1	8	0.28	0.6256
Habitat : Time	1	29	0.68	0.4165
Type : Time	1	29	0.05	0.8163
Habitat : Type : Time	1	29	0.82	0.3718

Table S9: Results of the mixed effect model analysis of the effect of habitat, polymer time, type, and their interactions on tensile strength. Three nested random effects were included to incorporate the structure of the nested design: 'Replicate' for the replicate construction (benthic and pelagic experimental systems), 'habnest' to account for the two samples per habitat (benthic vs. pelagic) and 'typenest' to account for the two samples of the two polymer types (PE vs. biodegradable plastic) per habitat.

Tensile strength	numDF	denDF	F-value	p-value
(Intercept)	1	55	1103.9407	<0.0001
Habitat	1	4	0.1754	0.6969
Type	1	8	4.2521	0.0731
Time	1	55	118.0894	<0.0001
Habitat :Type	1	8	0.4619	0.5159
Habitat : Time	1	55	1.7915	0.1863
Type : Time	1	55	96.9399	<0.0001
Habitat : Type : Time	1	55	0.1759	0.6765
